# Improving Immunohistochemistry Capability for Pediatric Cancer Care in the Central American and Caribbean Region: A Report From the AHOPCA Pathology Working Group

**DOI:** 10.1200/JGO.17.00187

**Published:** 2018-03-13

**Authors:** Teresa Santiago, Caleb Hayes, Ana Concepción Polanco, Lisa Miranda, Argelia Aybar, Belkis Gomero, Elizabeth Orellana, Fabienne Anglade, Mázlova Luxely Toledo González, Eduviges Ruiz, Moisés Espino-Durán, Carlos Rodriguez-Galindo, Monika L. Metzger

**Affiliations:** **Teresa Santiago**, **Caleb Hayes**, **Carlos Rodriguez-Galindo**, and **Monika L. Metzger**, St Jude Children’s Research Hospital, Memphis, TN; **Ana Concepción Polanco**, Hospital Nacional de Niños Benjamín Bloom, San Salvador, El Salvador; **Lisa Miranda**, Hospital Nacional de Niños “Dr. Carlos Sáenz Herrera,” San José, Costa Rica; **Argelia Aybar**, MediPath, Santiago City; **Belkis Gomero**, Hospital Infantil Dr. Robert Reid Cabral, Santo Domingo, Dominican Republic; **Elizabeth Orellana**, Francisco Marroquín Medical School, Guatemala City, Guatemala; **Fabienne Anglade**, Laboratory Regional Stephen at Pilar Robert, Mirebelais, Haiti; **Mázlova Luxely Toledo González**, Hospital Escuela-Universitario, Tegucigalpa, Honduras; **Eduviges Ruiz**, Hospital Infantil Manuel de Jesus Rivera “La Mascota,” Managua, Nicaragua; **Moisés Espino-Durán**, Hospital del Niño Dr. José Renán Esquivel, Panama City, Panama.

## Abstract

Accessibility to immunohistochemistry (IHC) is invaluable to proper diagnosis and treatment of pediatric patients with malignant neoplasms. Whereas IHC is widely available in anatomic pathology laboratories in high-income countries, access to it in anatomic pathology laboratories of low- and middle-income countries remains a struggle, with many limitations. To advance the quality of the pathology service offered to children with cancer in areas with limited resources, a 5-day pathology training workshop was offered to pathologists and histotechnologists from various countries of the Central American and Caribbean region. An initial assessment of the workshop participants’ current laboratory capacities was performed, and a regional training center was selected. Didactic and hands-on activities were offered, and review and evaluation of the IHC slides produced during the training course were compared with original slides from the participants’ sites. This model of intensive 5-day training appears to be effective and can potentially be used in other budget-constrained regions. Moreover, it can serve as a continuing education activity for pathologists and histotechnologists, and as part of validations and quality improvement projects to build capacity and develop IHC assay proficiency in low- and middle-income countries.

## INTRODUCTION

The use of immunohistochemistry (IHC) staining has long been recognized as an indispensable tool in surgical pathology practices to aid in the correct diagnosis and subclassification of the malignant neoplasms.^1,2^ An accurate diagnosis guides the clinical decision for proper treatment and consequently improves patients’ outcome. Although IHC is readily available and performed in most of the anatomic pathology (AP) laboratories in the developed world, significant limitations and even complete lack of IHC capabilities continue to exist in many AP laboratories in low- and middle-income countries (LMICs).

Since 1998, the pediatric oncology centers in Central America, later joined by the Caribbean countries of Haiti and the Dominican Republic, have formed the Associación de Hemato-Oncología Pediátrica de Centro América (AHOPCA). In collaboration with St Jude Children Research Hospital (SJCRH; Memphis, TN) and other institutions in North America and Europe, the AHOPCA group has promoted multidisciplinary educational activities and established shared clinical guidelines that have generated successful results through the years.^3-14^ Unfortunately, a significant gap and disparity in the level of the pathology services offered among these countries still exist, leading local oncologists and pathologists to request frequent second opinions from SJCRH. A previous analysis of cases submitted to SJCRH in consultation have shown a high index of incorrect diagnosis in the region, and the lack or poor quality of IHC assays was assumed to be one of the contributing factors.^15^

As part of a recently awarded P30 Cancer Center supplement (Grant No. 3P30CA021765-37S2) to promote clinical research studies in Burkitt lymphoma in LMICs, the pathologists from the Central America and Caribbean region started to participate in AHOPCA more actively and established the AHOPCA Pathology Working group (AHOPCA–Path). Whereas many factors contribute to the gap and disparity in the level of the pathology services offered in LMICs, we believe educational activities can be a powerful tool for capacity building and can consequently minimize the gap seen in these countries. As part of our effort to improve the quality of the pathology services offered to children in the region who have cancer, we promoted a 5-day pathology training workshop that focused on IHC. This workshop was provided not only to pathologists but also to histotechnologists. We believe this initiative can improve the technical and specialized knowledge of the participants, and help pathologists and histotechnologists make more appropriate decisions (including cost-saving choices) yet be able to implement proper standards and procedures. This approach may also be used as a model of training, capacity building, and to further develop IHC assay proficiency in other areas of the world with limited resources. Furthermore, local capacity training will lead to diagnostic independence and reduce the burden of second-opinion consultations while empowering the local pediatric oncology units and, ultimately, improve the outcomes of children with cancer.

## THE CURRENT STATE

The main characteristics of the AP laboratories from the AHOPCA member institutions are outlined in Table 1. Some AP laboratories in this area demonstrate overall good quality. However, a significant inequality in the infrastructure and capability of the AP laboratories is noted, ranging from private and well-equipped laboratories with existing automated IHC assays to public (institutional) laboratories that are restricted only to morphologic examination of hematoxylin and eosin (H&E)–stained slides and struggle with limitations in laboratory supplies imposed by economic restrictions. For many years, SJCRH has been offering second-opinion pathology diagnoses to the AHOPCA group. We have noticed that one of the leading reasons to submit a case in consultation is the impossibility of the local pathologists to further classify a neoplastic process because of the lack of IHC or poor IHC quality. Also, inadequate tissue fixation and suboptimal histologic sections are common issues that can substantially affect the ability to reach the correct diagnosis. Lack of quality-control activities and inappropriate IHC antibody optimization and validation were seen among some of the participants’ centers.

**Table 1:**
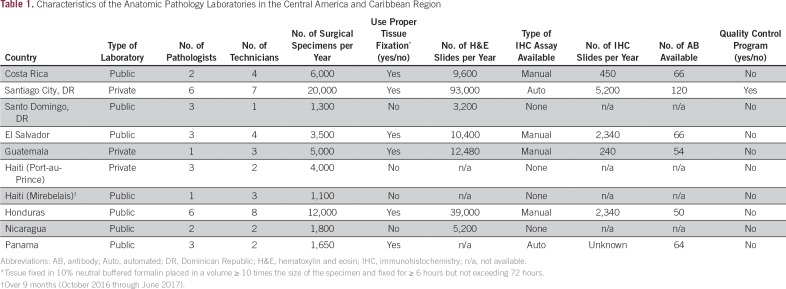
Characteristics of the Anatomic Pathology Laboratories in the Central America and Caribbean Region

During the workshop, all the pathologists were asked to give a 30-minute presentation using a previously provided template. The participants’ presentations helped delineate their current laboratory status, outline their assets, deficiencies, opportunities to improve, and potential threats (aka, SWOT analysis).^16^ This approach served not only as a self-assessment but additionally to highlight common problems among the centers (Table 2), encourage collaboration, and stimulate interaction among the AHOPCA–Path members. We believe establishing a strong regional network is an essential step toward improving the overall quality of the pathology service provided in this region, which will positively affect treatment and outcome of children with cancer.

**Table 2:**
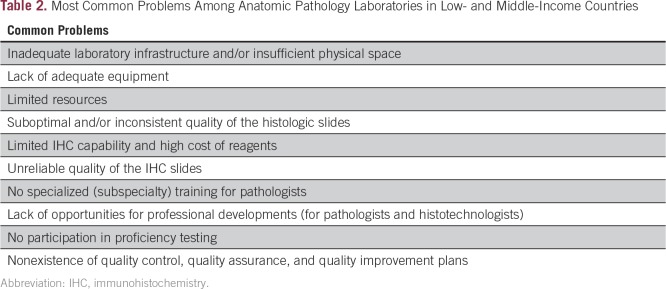
Most Common Problems Among Anatomic Pathology Laboratories in Low- and Middle-Income Countries

## THE TRAINING COURSE

### Training Center

**Fig 1:**
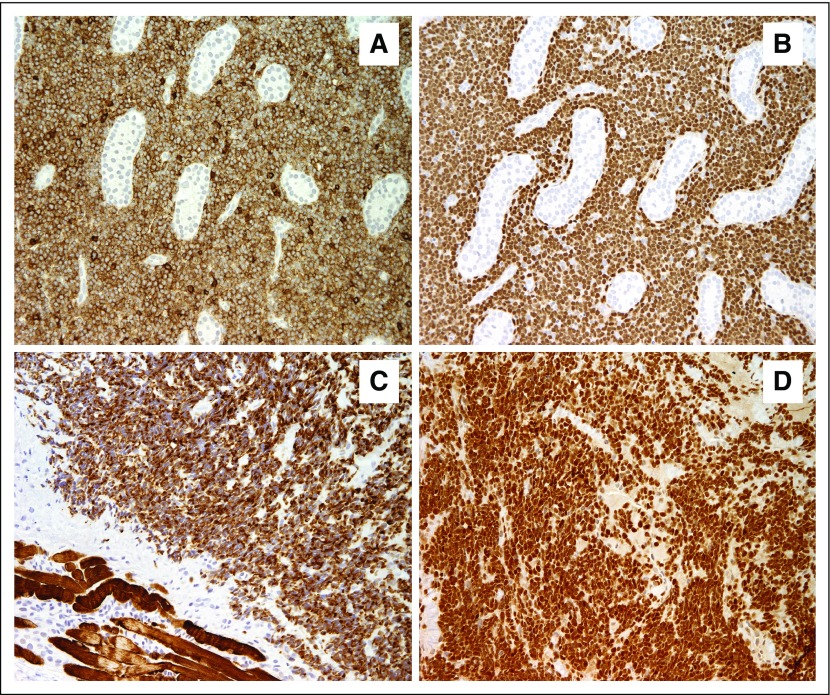
Micrographs of sample slides from the immunohistochemistry assay performed by hand at Hospital Nacional de Niños Benjamín Bloom, San Salvador, El Salvador. (A, B) Testis involved by lymphoblastic lymphoma, which is immunoreactive for (A) CD45 and (B) terminal deoxynucleotidyl transferase. (C, D) Tissue slides from a case of rhabdomyosarcoma that show diffuse positivity for (C) desmin and (D) Myo-D1. Magnification, ×200.

The Hospital Nacional de Niños Benjamín Bloom (HNNBB) in San Salvador, El Salvador, was strategically selected as the regional training center, and all the practice and didactic sessions occurred at HNNBB’s Department of Pathology. The HNNBB is a public, governmental, general pediatric hospital with 450 hospital beds. The Department of Pathology is institutional and is located within the main hospital. The pathology staff comprises three senior pathologists, four histotechnologists (two of them trained in IHC), four pathology assistants, and one administrative assistant. The laboratory was recently renovated, operates in a space of 250 m^2^, and is well equipped. The available equipment includes an automated tissue processor, a tissue embedding center, microtomes, a tissue water bath, an incubator, microscopes (including double headed), fume hoods, a cryostat, autopsy tables, a turbo mixer, micropipettes, and precision and analytical balances. At HNNBB, the IHC assay is performed by hand, on demand, and, based on a previous assessment, it demonstrates an excellent overall quality (Fig 1).

### Participants and Training Team

A total of 16 participants from Costa Rica, the Dominican Republic (Santiago and Santo Domingo), Guatemala, Haiti, Honduras, Nicaragua, and Panama attended this training workshop. Two members from each program (one pathologist and one histotechnologist) were invited. Some of the participants have experience in performing IHC by hand or have used automated IHC machines in their laboratories. Nevertheless, members from three centers (ie, Nicaragua, Haiti, and Santo Domingo in Dominican Republic) had never performed or used IHC in their daily practices. The training team was composed of two histotechnologists and one pathologist from HNNBB (A.C.P.) and a pathologist from SJCRH (T.S.).

### Didactic and Practice Activities

An overview of IHC concepts, including antigen-antibody reaction, specificity, control samples, antibody selection, and antibody optimization and validation was given during the educational sessions. There are many advantages to using an automated IHC over manual IHC staining; in particular, the fact that it can facilitate standardization and decrease the number of histotechnologists needed. Nevertheless, the high cost of acquiring and maintaining an automated IHC staining machine can be impracticable for many AP laboratories in LMICs. Therefore, our goal was to identify a center that had a well-established, high-quality, manual IHC assay that could be replicated in other centers where automated IHC staining could not be implemented. The practice sessions during the workshop for manual IHC staining followed the techniques currently in use at HNNBB (Table 3). Methods for proper tissue fixation, tissue processing, and appropriate tissue sectioning also were reviewed during the hands-on activities. Strategies for IHC implementation, budgeting, supplies acquisition, tactics of cost reduction, and troubleshooting of the IHC assay were discussed.

**Table 3:**
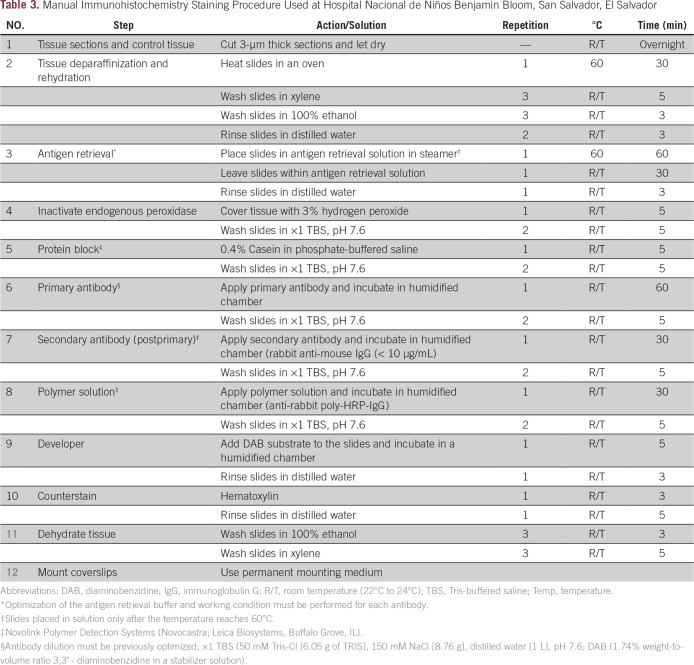
Manual Immunohistochemistry Staining Procedure Used at Hospital Nacional de Niños Benjamin Bloom, San Salvador, El Salvador

The workshop participants were asked to bring three paraffin blocks from three different cases of non-Hodgkin lymphoma that had been fixed and processed at their local institutions, as well as the corresponding H&E–stained slides and prestained IHC slides (if available). This material was deidentified and used during the practice sections. Pretreatment using a heat-induced epitope retrieval technique was performed, and slides were immunostained using commercially available antibodies. Anti-CD3 (Novocastra Catalog CD3-565-L-CE), anti-CD20 (Novocastra Catalog CD20-L26-L-CE), and anti-terminal deoxynucleotidyl transferase (Novocastra Catalog TdT-339-L-CE) had been previously selected for use during this workshop. The slides prepared during the workshop were compared with the original H&E–stained and IHC slides (if available) that had been prepared at their local institution using the same paraffin blocks.

All the slides stained during the workshop as well as the original slides were reviewed during the assessment sessions by the training team and the attendees using a microscope camera connected to a screen. In selected situations, individualized review (ie, an instructor with a trainee) took place to assess any discordant or suboptimal results. General and personalized recommendations were presented to the participants. All the participants (ie, pathologists and histotechnologists) had the opportunity to perform the manual IHC assay, and some participants had the chance to repeat the reactions up to three times. Aspects of IHC interpretation, reporting, quality-control plan, and competence assessment were also emphasized during the workshop. All the activities (didactic and hands-on) were offered in Spanish. Handouts, copies of protocols, and pictures of the slides were provided to the participants.

### Slide Review

**Fig 2:**
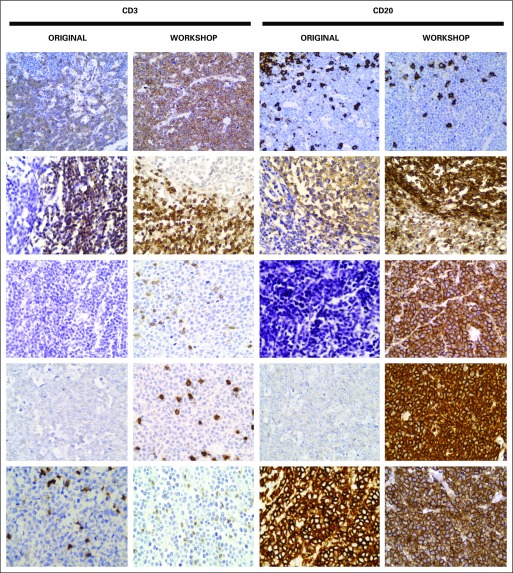
Parallel comparison of original immunohistochemistry slides from laboratories in five countries and slides stained during the workshop. Row 1: T-lymphoblastic lymphoma (sample from Costa Rica) showing similar excellent results, diffuse positivity for CD3 and negative for CD20, between original slides and slides stained during workshop. No background staining is noted. Row 2: Slides of lymphoid hyperplasia (sample from Santiago, Dominican Republic) were originally stained using an automated slide stainer and by hand during the workshop. These show comparable results with positivity for CD3 in the *para*-follicular region and CD20 immunoreactivity in the germinal centers. Row 3: Burkitt lymphoma (sample from Guatemala). Both original slides stained by hand (CD3 and CD20) show false-negative results. The same sample, when stained during the workshop, shows diffuse positivity for CD20 while negative for CD3, but with good internal control. Row 4: Burkitt lymphoma (sample from Honduras). The slide, originally stained by hand, shows only faint background staining. The same sample restained during the workshop after appropriate antigen retrieval shows proper diffuse positivity for CD20 and is negative for CD3 (with adequate positive internal control). Row 5: This Burkitt lymphoma sample slide (from Panama) displays diffuse positivity for CD20 and nonreactivity for CD3. A comparison between the original slides (stained using an automated slide stainer) and the slides stained by hand during the workshop show equivalent results. Magnification, ×400.

**Fig 3:**
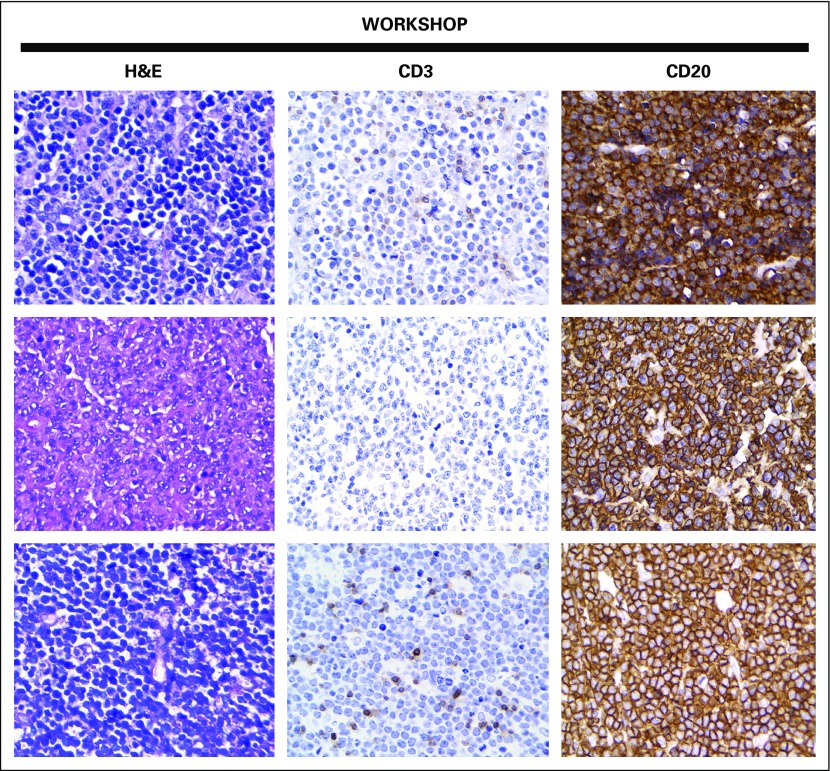
Micrographs from three cases of Burkitt lymphoma. H&E–stained slides reveal suboptimal tissue fixation. All the immunohistochemistry slides were stained manually during the workshop by histotechnologists without previous experience, and they show adequate results. All three cases were immunoreactive for CD20 and negative for CD3. (Row 1, sample from Santo Domingo; row 2, sample from Mirebalais, Haiti; row 3, sample from Nicaragua). H&E, hematoxylin and eosin. Magnification, ×400.

The evaluation of the H&E–stained slides enabled identification of problems with tissue fixation and/or with processing of the specimens. Four of the 10 centers (40%) did not routinely use 10% neutral buffered formalin nor did they monitor pH or fixation time before the workshop. IHC assays were already in use in six centers (including the training center): two of them use an automated IHC staining technique (Santiago in Dominican Republic, and Panama) and four centers perform IHC by hand (Costa Rica, Guatemala, Honduras, and El Salvador). Poor antigen retrieval, nonspecific staining, and intense background staining were examples of problems identified in some of the original IHC slides. During the workshop, all the slides were stained by hand, and the results were similar to the slides stained with an automated IHC stainer. A parallel comparison of the original slides and slides stained during the workshop is presented in Figs 2 and 3.

### Summary

Based on the assessment of the participants’ performance during the workshop, the evaluation of their original slides, and the slides prepared during the training sessions, we believe this model of intensive 5-day training with a combination of didactic and practice activities appears to be a useful strategy to improve IHC capacity in countries with limited resources. Nevertheless, the effect of this training workshop will need to be evaluated with short- and long-term follow-up evaluations to appraise any postworkshop changes and progress. We also believe promoting regular communication and collaboration among the participants is critical to allow regional development.

In summary, this 5-day workshop showed that a high-quality IHC assay performed by hand in a limited resource setting is achievable. When well controlled, the results of IHC assay done by hand can be reproducible and the overall performance similar to the staining obtained with an automated IHC stainer. Inadequate tissue fixation and processing, which can compromise the tissue sample interpretation and final diagnosis were identified in some participant laboratories. Strategies to improve tissue fixation and processing were addressed during the training sessions. Proper documentation, standardization, and quality-control activities were nonexistent in the vast majority of the participating institutions. The implementation and daily use of appropriate standards and procedures, and quality monitors can ensure high-quality results and reproducibility of the IHC assay in areas with limited resources. Moreover, we believe this model of training can be replicated in other LMICs.

## References

[1] Parham DM (2015). Immunohistochemical markers of soft tissue tumors: Pathologic diagnosis, genetic contributions, and therapeutic options. Anal Chem Insights.

[2] Sebire NJ, Gibson S, Rampling D (2005). Immunohistochemical findings in embryonal small round cell tumors with molecular diagnostic confirmation. Appl Immunohistochem Mol Morphol.

[3] Sala A, Antillon F, Pencharz P (2005). Nutritional status in children with cancer: A report from the AHOPCA Workshop held in Guatemala City, August 31-September 5, 2004. Pediatr Blood Cancer.

[4] Howard SC, Marinoni M, Castillo L (2007). Improving outcomes for children with cancer in low-income countries in Latin America: A report on the recent meetings of the Monza International School of Pediatric Hematology/Oncology (MISPHO)-Part I. Pediatr Blood Cancer.

[5] Antillon F, de Maselli T, Garcia T (2008). Nutritional status of children during treatment for acute lymphoblastic leukemia in the Central American Pediatric Hematology Oncology Association (AHOPCA): Preliminary data from Guatemala. Pediatr Blood Cancer.

[6] Sala A, Rossi E, Antillon F (2008). Nutritional status at diagnosis in children and adolescents with cancer in the Asociacion de Hemato-Oncologia Pediatrica de Centro America (AHOPCA) countries: Preliminary results from Guatemala. Pediatr Blood Cancer.

[7] Sala A, Rossi E, Antillon F (2012). Nutritional status at diagnosis is related to clinical outcomes in children and adolescents with cancer: A perspective from Central America. Eur J Cancer.

[8] Luna-Fineman S, Barnoya M, Bonilla M (2012). Retinoblastoma in Central America: Report from the Central American Association of Pediatric Hematology Oncology (AHOPCA). Pediatr Blood Cancer.

[9] Friedrich P, Ortiz R, Strait K (2013). Pediatric sarcoma in Central America: Outcomes, challenges, and plans for improvement. Cancer.

[10] Friedrich P, Ortiz R, Fuentes S (2014). Barriers to effective treatment of pediatric solid tumors in middle-income countries: Can we make sense of the spectrum of nonbiologic factors that influence outcomes?. Cancer.

[11] Castellanos EM, Barrantes JC, Báez LF (2014). A chemotherapy only therapeutic approach to pediatric Hodgkin lymphoma: AHOPCA LH 1999. Pediatr Blood Cancer.

[12] Navarrete M, Rossi E, Brivio E (2014). Treatment of childhood acute lymphoblastic leukemia in Central America: A lower-middle income countries experience. Pediatr Blood Cancer.

[13] Barr RD, Antillón Klussmann F, Baez F (2014). Asociación de Hemato-Oncología Pediátrica de Centro América (AHOPCA): A model for sustainable development in pediatric oncology. Pediatr Blood Cancer.

[14] Ceppi F, Ortiz R, Antillón F (2016). Anaplastic large cell lymphoma in Central America: A report from the Central American Association of Pediatric Hematology Oncology (AHOPCA). Pediatr Blood Cancer.

[15] Santiago TC, Jenkins JJ (2013). Histopathologic diagnosis of pediatric neoplasms: A review of international consultations. Arch Pathol Lab Med.

[16] Casebeer A (1993). Application of SWOT analysis. Br J Hosp Med.

